# Long-term remission and survival in patients with relapsed or refractory multiple myeloma after treatment with LCAR-B38M CAR T cells: 5-year follow-up of the LEGEND-2 trial

**DOI:** 10.1186/s13045-024-01530-z

**Published:** 2024-04-24

**Authors:** Jie Xu, Bai-Yan Wang, Shan-He Yu, Shi-Jun Chen, Shuang-Shuang Yang, Rui Liu, Li-Juan Chen, Jian Hou, Zhu Chen, Wan-Hong Zhao, Ai-Li He, Jian-Qing Mi, Sai-Juan Chen

**Affiliations:** 1 State Key Laboratory of Medical Genomics, National Research Center for Translational Medicine, Shanghai Institute of Hematology, Ruijin Hospital affiliated with Shanghai Jiao Tong University School of Medicine, 197 Rui Jin Er Road, Shanghai, 200025 China; 2 Department of Hematology, Second Affiliated Hospital of Xi’an Jiao Tong University, 157 West 5th Road, Xi’an, 710004 China; 3 Department of Hematology, Jiangsu Province Hospital, First Affiliated Hospital of Nanjing Medical University, Nanjing, 210029 China; 4 Department of Hematology, Renji Hospital affiliated with Shanghai Jiao Tong University School of Medicine, Shanghai, 200127 China

**Keywords:** LCAR-B38M, CAR T-cell therapy, Relapsed and refractory multiple myeloma, Long-term follow-up, Long-persisting CAR T cell

## Abstract

**Background:**

The autologous anti–B-cell maturation antigen (BCMA) chimeric antigen receptor (CAR) T-cell therapy LCAR-B38M has been approved for the treatment of relapsed and refractory multiple myeloma in many countries across the world under the name ciltacabtagene autoleucel. LEGEND-2 was the first-in-human trial of LCAR-B38M and yielded deep and durable therapeutic responses. Here, we reported the outcomes in LEGEND-2 after a minimal 5-year follow-up.

**Methods:**

Participants received an average dose of 0.5 × 10^6^ cells/kg LCAR-B38M in split or single unfractionated infusions after cyclophosphamide-based lymphodepletion therapy. Investigator-assessed response, survival, safety and pharmacokinetics were evaluated.

**Results:**

Seventy-four participants enrolled and had a median follow-up of 65.4 months. The 5-year progression-free survival (PFS) and overall survival (OS) rates were 21.0% and 49.1%, with progressive flattening of the survival curves over time. Patients with complete response (CR) had longer PFS and OS, with 5-year rates of 28.4% and 65.7%, respectively. Twelve patients (16.2%) remained relapse-free irrespective of baseline high-risk cytogenetic abnormality and all had normal humoral immunity reconstituted. An ongoing CR closely correlated with several prognostic baseline indices including favorable performance status, immunoglobulin G subtype, and absence of extramedullary disease, as well as a combination cyclophosphamide and fludarabine preconditioning strategy. Sixty-two (83.8%) suffered progressive disease (PD) and/or death; however, 61.1% of PD patients could well respond to subsequent therapies, among which, the proteasome inhibitor-based regimens benefited the most. Concerning the safety, hematologic and hepatic function recovery were not significantly different between non-PD and PD/Death groups. A low rate of second primary malignancy (5.4%) and no severe virus infection were observed. The patients who tested positive for COVID-19 merely presented self-limiting symptoms. In addition, a sustainable CAR T population of one case with persistent remission was delineated, which was enriched with indolently proliferative and lowly cytotoxic CD4/CD8 double-negative functional T lymphocytes.

**Conclusions:**

These data, representing the longest follow-up of BCMA-redirected CAR T-cell therapy to date, demonstrate long-term remission and survival with LCAR-B38M for advanced myeloma.

**Trial registration:**

LEGEND-2 was registered under the trial numbers NCT03090659, ChiCTRONH-17012285.

**Supplementary Information:**

The online version contains supplementary material available at 10.1186/s13045-024-01530-z.

## Background

The deep and durable responses with manageable safety profile of LCAR-B38M, also known as ciltacabtagene autoleucel (cilta-cel), were demonstrated in pivotal trials CARTITUDE-1 and CARTIFAN-1 for relapsed and refractory multiple myeloma (RRMM) [1, 2]. Based on the results of those trials, a number of countries and areas, such as the United States of America (USA), the European Union, Japan, South Korea, have granted marketing authorization to LCAR-B38M for the RRMM after three or four prior lines of treatment. As a subsequent step, studies of LCAR-B38M for different disease stages are being ongoing to further explore its efficacy in additional indications. The CARTITUDE-2 cohort B study (NCT04133636) has reported an overall response rate (ORR) of 100% in patients with early relapse after frontline therapy [3]. The latest data from the CARTITUDE-4 (NCT04181827) trial demonstrated superiority of cilta-cel over standard of care treatments in lenalidomide-refractory patients with one to three prior lines of therapy [4]. Owing to these remarkable advances, the US Food and Drug Administration recently approved cilta-cel as the first BCMA-targeted treatment for RRMM who have received at least one prior line of therapy.

With the growing use of LCAR-B38M, the long-term survival of the CAR T-exposed patients garners increased attention. The follow-up information is needed for a comprehensive evaluation of product quality and a clear direction of scheme optimization. Meanwhile, since CAR T cell emerges as a paradigm shift of treatment in myeloma, whether such an innovative modality could offer a cure hasn’t been clearly demonstrated. The phase I first-in-human trial of LCAR-B38M, LEGEND-2 study (NCT03090659, ChiCTRONH-17012285), was initiated by four medical centers in China 7 years ago [5, 6]. At a median 4-year follow-up, median progression-free survival (PFS) was 18 months, and median overall survival (OS) had not been reached [7]. We now report on the results at ≥ 5-year follow-up.

In the present study, at a median follow-up of half-decade long, 16.2% of participants in the LEGEND-2 had durable responses and still maintained relapse-free. The longest remission had been 6.4 years. One patient who achieved a 5-year deep response persistently had LCAR-B38M in circulation. The updates and new reporting strongly demonstrate LCAR-B38M’s potent anti-tumor effect in advanced myeloma.

## Methods

### Study procedure

The LEGEND-2 study (NCT03090659, ChiCTRONH-17012285) was conducted at four clinical centers in China: Second Affiliated Hospital of Xi’an Jiao Tong University (Xi’an), Ruijin Hospital affiliated with Shanghai Jiao Tong University (RJ), First Affiliated Hospital of Nanjing Medical University (NJ), and Changzheng Hospital (CZ). Experiments were performed in accordance with the study protocol, which was approved by the review board of the ethics committee at each site. All patients provided their written informed consent.

Preconditioning chemotherapy was given for three consecutive days to deplete host lymphocytes. The regimen administered to the patients at the Xi’an and NJ sites was a single agent cyclophosphamide (300mg/m^2^/day×3 days), whereas the regimen administered for RJ and CZ consisted of cyclophosphamide (250mg/m^2^/day×3 days) and fludarabine (25mg/m^2^/day×3 days). After a two-day interval, LCAR-B38M was intravenously administrated with a total dose of 0.5 × 10^6^/kg at average, either as a single dose (NJ) or split doses (Xi’an, RJ, and CZ). The latter infusion mode required patients to receive 20%, 30%, and 50% of the total CAR T cell dose on day 0, 2, and 6, respectively. Starting from the day 0, close monitoring, essential tests and regular management were performed to prevent and treat CAR T-associated toxicity. The procedure was schematically displayed in Supplementary Fig. 1.

### Long-term outcome assessments

Drug response was assessed in accordance to the International Myeloma Working Group consensus criteria [8]. Survival status was confirmed at the last follow-up. For the surviving patients, subsequent therapies information was obtained from medical records and routine disease evaluation results including serum/urine immunofixation electrophoresis, bone marrow (BM) aspiration/biopsy, PET-CT were reviewed by physicians. Baseline demographic data were retrieved from the patient enrollment and treatment databases preserved by the LEGEND-2 study group.

Peripheral blood cells counts and hepatic function were obtained from medical records. Any incidence of infectious diseases, including COVID-19, was documented through phone interviews and accessible records. Replication-competent lentivirus reports were provided by Legend Biotech. Other late safety events, such as secondary tumor and second primary malignancy (SPM), were collected from medical records.

Minimal residual disease (MRD) was assessed by flow cytometry, using a ten-color panel of antibodies against human CD45 (APC-H7), CD38 (BV510), CD138 (APC), CD19 (PE-CY7), CD56 (APC-A700), CD117 (PE-CY5.5), CD81 (PB), CD27 (BV605), cytoplasmic kappa (FITC), and cytoplasmic lambda (PE). Kappa and lambda antibodies were purchased from Dako (Carpinteria, CA), CD81 and CD138 were supplied by Beckman Coulter (Brea, California), and the other six antibodies were purchased from BD (San Jose, California). The antibody-labeled BM nucleated cells were run on FACSCanto™ flow cytometer (Becton Dickinson, San Jose, CA, USA). Data was analyzed using KALUZA software (Beckman Coulter, Brea, California). MRD was defined as a distinct cluster of at least 30 plasma cells carrying aberrant immunophenotypes. At least 1.5 million nucleated cells for each sample were acquired to reach a sensitivity threshold of 1.0 × 10^− 4^. MRD was quantified as a percentage of total white blood cells after exclusion of doublets, debris, and erythrocytes. Any level of an abnormal plasma cell population was considered as MRD positive.

### Mass cytometry (CyTOF)

A panel of metal-conjugated antibodies was used, including an anti-camelid variable domain of heavy chain of heavy-chain (VHH) antibody for tracing CAR T cells. Metal-coated antibodies were prepared using the Maxpar X8 Antibody Labeling Kit (Fluidigm) according to the manufacturer’s instructions.

Sample preparation, antibody staining, data acquisition, readout processing were previously described [9]. In brief, cryopreserved samples were rapidly thawed and assayed for cell number and viability. Cells were stained with Cisplatin (Fluidigm), resuspended in Cell Staining Buffer (Fluidigm), and incubated with Human TruStain FcX (Biolegend). Each sample was subsequently labeled with cell surface antibody cocktail. After washing, samples were fixed in paraformaldehyde followed by treatment with the DNA intercalator (Fluidigm). The stained cells were resuspended in freshly prepared Cell Acquisition Solution (Fluidigm). Data were subsequently acquired on CyTOF in National Research Center for Translational Medicine (Shanghai). Data of individual sample were manually gated using Cytobank for downstream analysis. t-SNE and PhenoGraph algorithm were performed on all samples. Data were displayed using the ggplot2 R package.

### Statistical analysis

Two-sided 95% exact confidence intervals (CIs) based on binomial distribution were calculated for each response category. Median PFS, OS and duration of response (DOR), and corresponding 95% CIs were calculated using Kaplan-Meier methods. Clinical response comparison by diverse parameters was made by a Log-rank test or a Wilcoxon rank-sum test. A Chi-square test was applied for comparing proportions of a categorical outcome among different groups.

## Results

### Clinical characteristics and disposition

A total of 74 RRMM patients were enrolled. Patients had previous exposure to alkylating agents and/or anthracycline-containing chemotherapy (93.2%) and proteasome inhibitors (PIs) and/or immunomodulatory drugs (IMiDs) (95.9%), while 24.3% of patients underwent autologous hematopoietic stem cell transplantation (Supplementary Table 1). The median number of prior lines of therapy was three (range 1∼9). Patients aged 27∼74 years old with a median of 54.5. 16.2% of participants had poor Eastern Cooperative Oncology Group (ECOG) performance status (≥ 2 scores). Immunoglobulin (Ig) G (44.6%) subtype accounted for the most, followed by IgA (28.4%) and light chain (25.7%). With respect to tumor burden and myeloma biology, 28.4% of the cases were stratified as International Staging System (ISS) III and 75.7% were categorized as Durie-Salmon System III. Extramedullary plasmacytoma was present in 29.7% of patients at baseline. Cytogenetic abnormalities (CA) information including t(4;14), t(14;16), t(14;20), del(17p), and gain(1q), was available in 42 patients, 78.6% of whom bore high-risk CA. According to the updated risk stratification proposed by the Mayo clinic [10], 23.8% and 2.4% of the 42 patients were involved with double-hit and triple-hit diseases, respectively. The mean level of serum β2-microglobulin was 5.8 mg/L. Details are shown in Table 1.

**Table 1:** Clinical relevance of baseline demographics

Parameters	Total	Non-PD	PD/Death	*p* value
Number of patients	74	12	62	
**Host Characteristics**				
Age (years)				
Median (range)	54.5 (27 ∼ 74)	52.5 (35 ∼ 68)	55.0 (27 ∼ 74)	0.5654
ECOG performance status (n)				
0	30 (40.5%)	8 (66.7%)	22 (35.5%)	0.0144
1	32 (43.2%)	1 (8.3%)	31 (50.0%)	
2	12 (16.2%)	3 (25.0%)	9 (14.5%)	
Myeloma subtype (n)				
IgG	33 (44.6%)	9 (75.0%)	24 (38.7%)	0.0206
IgA	21 (28.4%)	3 (25.0%)	18 (29.0%)	1.0000
IgD	1 (1.4%)	0	1 (1.6%)	1.0000
Kappa	7 (9.5%)	0	7 (11.3%)	0.5901
Lambda	12 (16.2%)	0	12 (19.4%)	0.1953
**Tumor Burden**				
ISS staging (n)				
I	33 (44.6%)	8 (66.7%)	25 (40.3%)	0.3824
II	14 (18.9%)	1 (8.3%)	13 (21.0%)	
III	21 (28.4%)	3 (25.0%)	18 (29.0%)	
Unknown	6 (8.1%)	0	6 (9.7%)	
Durie-Salmon system (n)				
IIA	8 (10.8%)	2 (16.7%)	6 (9.7%)	0.5751
IIB	1 (1.4%)	0	1 (1.6%)	
III^§^	3 (4.1%)	0	3 (4.8%)	
IIIA	44 (59.5%)	7 (58.3%)	37 (59.7%)	
IIIB	9 (12.2%)	0	9 (14.5%)	
Unknown	9 (12.2%)	3 (25.0%)	6 (9.7%)	
Extramedullary plasmacytoma (n)				
Yes	22 (29.7%)	0	22 (35.5%)	0.0139
No	52 (70.3%)	12 (100.0%)	40 (64.5%)	
**Tumor biology**				
Cytogenetic abnormalities (n)	42	8	34	
Standard risk	9 (21.4%)	1 (12.5%)	8 (23.5%)	0.6622
High risk	33 (78.6%)	7 (87.5%)	26 (76.5%)	
t(4;14)	5 (11.9%)	1 (12.5%)	4 (11.8%)	
t(14;16)	0	0	0	
t(14;20)	0	0	0	
del(17p)	11 (26.2%)	2 (25.0%)	9 (26.5%)	
gain(1q)	29 (69.0%)	7 (87.5%)	22 (64.7%)	
Double-hit^†^	10 (23.8%)	3 (37.5%)	7 (20.6%)	
Triple-hit^‡^	1 (2.4%)	0	1 (2.9%)	
Serum β2-microglobuline (mg/L)				
Mean (SD)	5.8 (4.7)	4.1 (2.4)	6.1 (4.9)	0.1039
Median (range)	3.9 (1.7 ∼ 31.7)	3.7 (1.9 ∼ 9.0)	4.3 (1.7 ∼ 31.7)	
**Previous medical history**				
Time from initial MM diagnosis (years)				
Mean (SD)	3.9 (2.1)	3.5 (2.2)	4.0 (2.0)	0.3445
Median (range)	4.0 (1 ∼ 9)	3.0 (1 ∼ 9)	4.0 (1 ∼ 9)	
Number of prior lines of therapy (n)				
Mean (SD)	3.2 (1.8)	2.8 (1.5)	3.3 (1.8)	0.2797
Median (range)	3.0 (1 ∼ 9)	2.0 (1 ∼ 6)	3.0 (1 ∼ 9)	
**CAR T treatment scheme**				
Total CAR T cells (×10^6^/kg)				
Median (range)	0.5 (0.1–2.1)	0.4 (0.2–1.6)	0.5 (0.1–2.1)	0.7204
Infusion mode (n)				
Unfractionated infusion	9 (12.2%)	0	9 (14.5%)	0.3392
fractionated infusion	65 (87.8%)	12 (100.0%)	53 (85.5%)	
Preconditioning therapy (n)				
Cyclophosphamide	66 (89.2%)	8 (66.7%)	58 (93.5%)	0.0204
Fludarabine + Cyclophosphamide	8 (10.8%)	4 (33.3%)	4 (6.5%)	

*Note* § The Duric-Salmon staging of the three patients could not be further stratified due to unavailability of serum creatinine levels at baseline. †,‡Multiple myeloma (MM) disease having any two of the high-risk cytogenetic abnormalities including t(4;14), t(14;16), t(14;20), del(17p), and gain(1q) is defined as double-hit MM and having any three as triple-hit MM. *P* values were calculated by t-test or Fisher’s exact test. CAR, Chimeric antigen receptor; ECOG, Eastern Cooperative Oncology Group; Ig, Immunoglobulin; ISS, International Staging System; PD, Progressive disease; SD, Standard deviation

### Clinical significance of patients’ characteristics

**Fig. 1:**
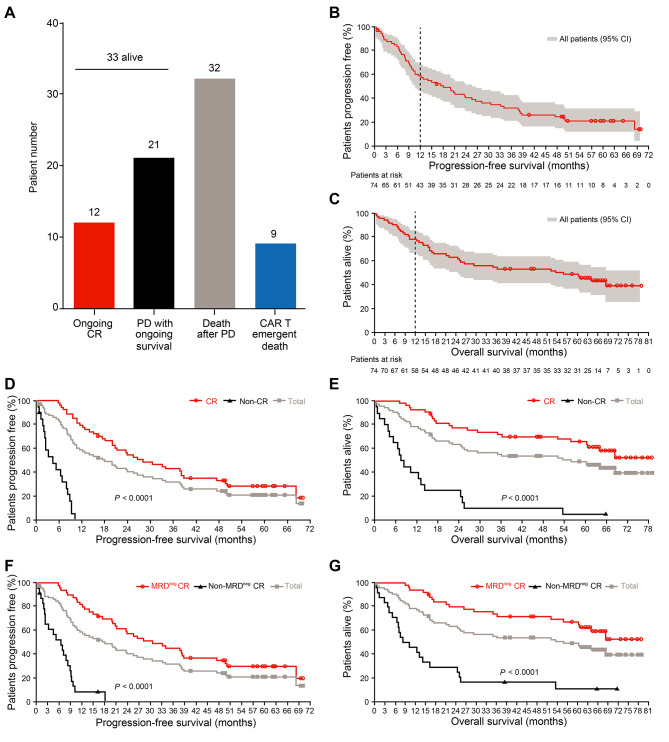
Long-term efficacy of LCAR-B38M. **A**) The column graph shows the number of patients with different survival states. CR: complete response; PD: progressive disease. (**B, C**) The Kaplan-Meier survival curves show the progression-free survival (PFS) rate (**B**) and the overall survival (OS) rate (**C**) of all 74 patients at a median follow-up time of 65.4 months. (**D, E**) The Kaplan-Meier survival curves compare the PFS (**D**) and OS (**E**) rates between the 54 patients achieving CR and the rest who did not have CR (Non-CR). (**F, G**) The Kaplan-Meier survival curves compare the PFS (**F**) and OS (**G**) rates between the two CR cohorts with (Non-MRD^neg^ CR) and without (MRD^neg^ CR) measurable residual disease (MRD), respectively

As previously reported [7], the ORR was 87.8% (65/74 patients). Fifty-four (73.0%) patients achieved complete response (CR), and 50 (67.6%) obtained deep remission with MRD negativity (< 10^− 4^). As of November 30, 2022, 33 (44.6%) patients were still alive. Notably, 12 (16.2%) remained relapse-free. The longest remission had been 6.4 years. Sixty-two patients suffered progressive disease (PD) and/or death (PD/Death). Among them, 53 appeared PD after partial response (PR) or better. Thirty-two died of PD and 9 were deceased owing to non-relapse events. Twenty-one PD patients had ongoing survival after receiving subsequent treatments (Fig. 1A).

Patients with durable responses were more likely to have favorable baseline performance status (ECOG score = 0), IgG MM subtype, and no extramedullary disease compared with those who had PD/Death. Patients with lambda light chain MM had a tendency toward inferior outcomes although no statistical difference was reached. In addition, high-risk CA was not a poor prognostic factor in this trial (Table 1). Data suggested that use of combined preconditioning strategy with fludarabine and cyclophosphamide may confer a therapeutic advantage over cyclophosphamide alone (Table 1).

### Long-term efficacy

With a median follow-up time of 65.4 months, the 5-year PFS rate was 21.0% (95% CI = 12.2%∼31.4%, Fig. 1B), and the 5-year OS rate was 49.1% (95% CI = 37.2%∼60.0%, Fig. 1C) across all treated patients. The PFS and OS curves progressively flattened over time. Among the 65 responders, the 5-year PFS and OS rates were 23.6% and 55.9%, respectively (Supplementary Fig. 2A and 2B). The survival status of the 54 CR patients was further improved. Their 5-year PFS and OS rates were respectively 28.4% and 65.7%, both being significantly higher than those without achieving CR (*p* < 0.0001, Fig. 1D and E). Such a meaningful benefit was more pronounced in the patients with MRD negativity relative to those with positivity (*p* < 0.0001, Fig. 1F and G). 24.0% (12/50) of the MRD-negative responders remained in deep remission with sustained responses at data cut off.

Median PFS and median OS respectively reached 18.0 and 55.8 months. For the subjects achieving CR and MRD-negative CR, the median PFS was prolonged to 28.2 and 30.6 months, respectively. Median OS was not reached for those patients (Supplementary Table 2 ∼ 5). Conversely, patients who did not obtain CR or better response showed inferior outcomes as they only had a median PFS of 4.4 months and a median OS of 7.9 months (Supplementary Tables 2 and 4). Median DOR for all patients was 23 months, and the MRD-negative responders displayed a significantly longer median DOR than the other responders (32.7 months versus 7.5 months, *p* < 0.0001, Supplementary Table 6). These data suggest that patients with an in-depth response are more likely to have favorable outcomes.

**Fig. 2:**
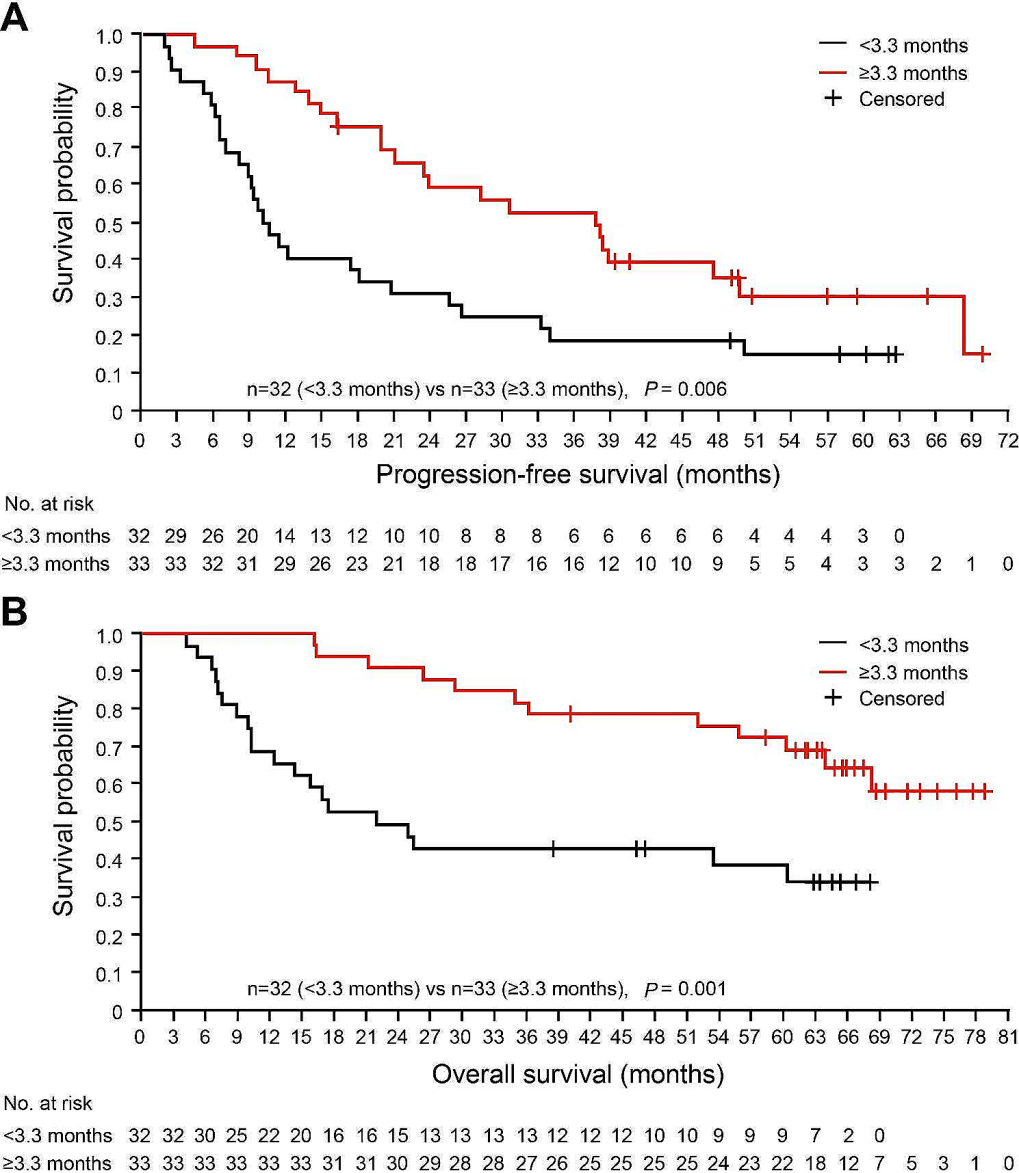
Outcomes by time to best response. (**A, B**) The Kaplan-Meier survival curves compare the progression-free survival (**A**) and the overall survival (**B**) rates between the patients who reached the best responses shorter and longer than 3.3 months after CAR T cell infusions

To evaluate if an early good response could lead to durable responsiveness, the relationship between the time to response and the remission persistence was assessed. It turned out that patients with longer time to best response (≥ 3.3 months) had longer PFS and OS compared with those achieving best response within 3.3 months (Fig. 2A and B). A late best response was associated with IgG subtype and higher ISS stage (Supplementary Table 7).

Normal Ig recovery represents a steady-state condition of humoral immunity for the patients who achieved remission. In 26 CR patients with available Ig data, 21 were found to have serologically normal Ig levels. Only one patient’s Ig level turned normal at 5.4 years and the rest twenty were recovered by 5 years (Supplementary Table 8). The median time to a full restoration was 16.7 months with the earliest emerging at 9.1 months post-infusion. All patients who were persistently relapse-free had serum Ig recovery (Supplementary Table 8), implying a completely resolved humoral immunity. The 5-year OS rate of the 21 patients with CR and a full Ig recovery was 100% but was not significantly different from those five patients who had abnormal levels, probably because of an unbalanced sample size (*p* = 0.14, Supplementary Fig. 3). Patients in CR with a normal Ig level did not report serious infections.

It is noteworthy that the risk of progression still existed after 5-year remission. Disease relapse occurred at 68.3, 69.5, and 62.4 months post-infusion in three patients who had achieved MRD-negative CR (Supplementary Table 9). One patient developed extramedually lesion without detectable tumor in BM, and two patients had an increased level of monoclonal paraprotein. All proceeded with subsequent therapies.

Of 53 patients who progressed, 44 received subsequent treatments, most commonly PIs, followed by IMiDs, BCMA-redirected CAR T cell products, and anti-CD38 monoclonal antibody (Table 2). Of 36 patients with response data, 22 (61.1%) patients were responsive to the post-CAR T salvage therapies, obtaining PR or better. PI-based regimen induced the highest ORR (80.0%) and the highest CR rate (55.0%). Salvage treatment with anti-BCMA CAR T infusions only benefited 31.3% patients, which was lower than the previous ORR of 87.8% (Table 2). Most patients who gained a second remission had maintenance treatment. Two patients discontinued treatment after obtaining CR with autologous hematopoietic stem cell transplantation or anti-BCMA CAR T intervention. Importantly, 21 patients survived more than two years additionally after PD.

**Table 2:** Subsequent therapies for patients who had progressive diseases after LCAR-B38M

Drugs	Number of patients	Number of patients with evaluable response	Overall response rate	CR rate
Total	44	36	61.1% (22/36)	38.9% (14/36)
Proteasome inhibitors	23	20	80.0% (16/20)	55.0% (11/20)
Bortezomib	18			
Carfilzomib	1			
Ixazomib	5			
Immunomodulatory drugs	21	19	63.2% (12/19)	36.8% (7/19)
Thalidomide	5			
Lenalidomide	12			
Pomalidomide	7			
Anti-CD38 mAb	9	7	71.4% (5/7)	42.9% (3/7)
Anti-BCMA CAR T cells	18	16	31.3% (5/16)	12.5% (2/16)

*Note* The therapeutic effect induced by two or more drugs in a combinatory regimen was separately analyzed in different drug categories. BCMA, B-cell maturation antigen; CAR, Chimeric antigen receptor; CR, Complete response; mAb, Monoclonal antibody

### Long-term safety

All patients experienced treatment-emergent adverse events (TEAEs) during the first month of treatment. Cytokine release syndrome (CRS) was the most common TEAE and occurred in 68 (91.9%) participants. This complication was mostly limited to low grade and all clinically reversible. CRS resolved within 30 days of CAR T cell infusion for most patients, but three patients had extended CRS which lasted 31, 37, and 51 days. No second wave of CRS was observed. Grade 3/4 neutropenia, thrombocytopenia, and hepatic disorder were observed in 85.3%, 58.8%, and 38.3% of patients, respectively. One patient experienced transient Grade 1 immune effector cell-associated neurotoxicity syndrome. One patient died of suicide after a psychiatric disorder that developed following myeloma progression; the patient had no abnormal parameters indicative of movement and neurocognitive TEAEs.

**Fig. 3:**
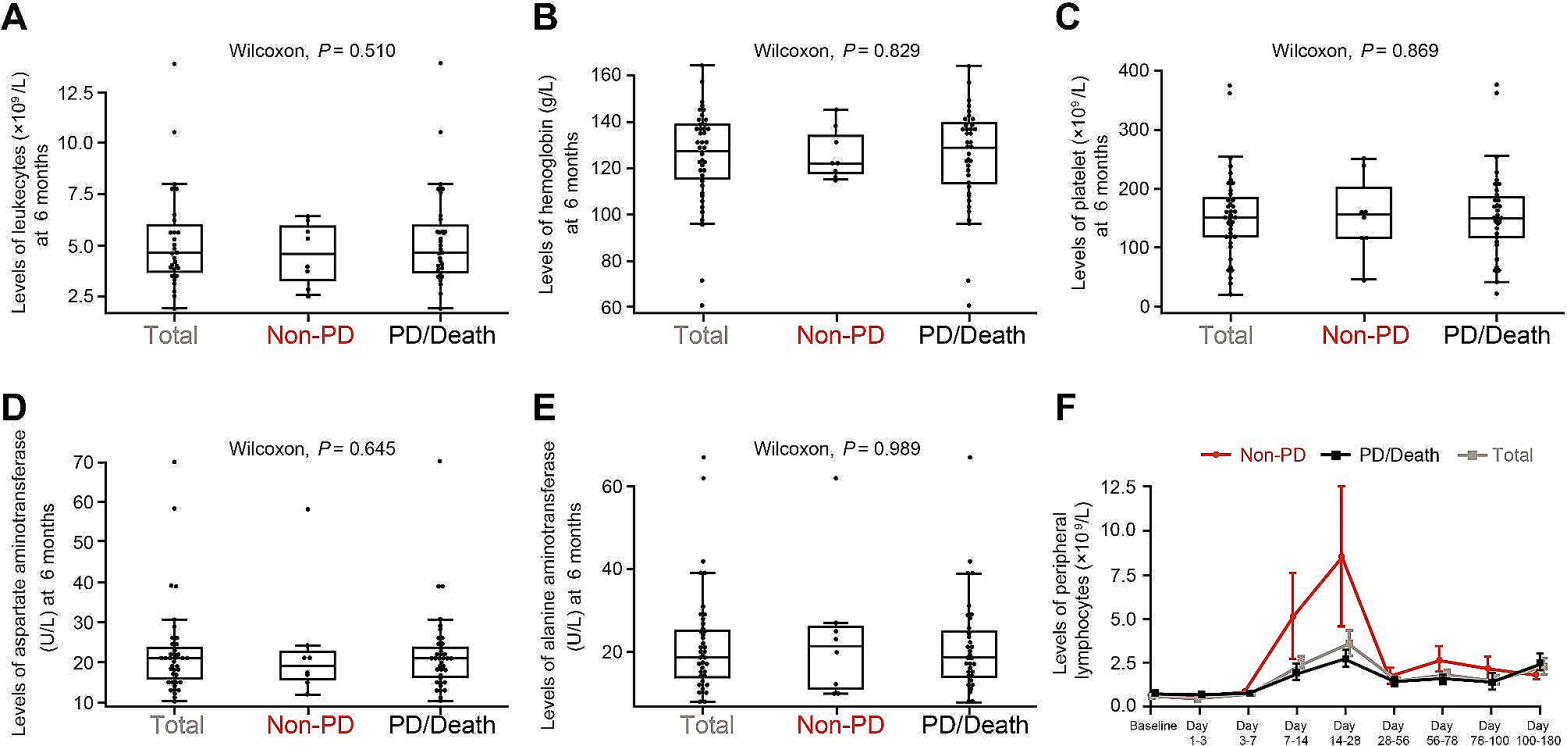
Hematologic and liver function recovery by 6 months post-infusion. (**A, B, C**) The box plots show the levels of leukocyte (**A**), hemoglobin (**B**) and platelet (**C**) of all patients (Total), the patients with ongoing response (Non-PD) and the patients having progression disease and/or being deceased (PD/Death). (**D, E**) The box plots show the levels of aspartate aminotransferase (**D**) and alanine aminotransferase (**E**) of all patients, as well as the patients with Non-PD and PD/Death. (**F**) The curves display the circulating lymphocyte counts of Non-PD, PD/Death and all patients

By 6 months post-infusion, 47.3%, 44.6% and 59.5% of all patients reached normal leukocyte, hemoglobin and platelet levels, respectively, and median levels were 4.7 × 10^9^/L, 125.3 g/L and 155.0 × 10^9^/L, respectively (Fig. 3A ∼ C). The median levels of aspartate aminotransferase and alanine aminotransferase were 21.0 U/L and 18.5 U/L, respectively (Fig. 3D and E). No significant differences in hematologic and hepatic function recovery were found between non-PD and PD/Death groups (Fig. 3A ∼ E), indicating that safety profile does not influence the patients’ efficacy outcomes.

Virus reactivation or infection was observed beyond 6 months of follow-up but no severe situation was reported. Herpes zoster virus involvement was documented in 3 patients and hepatitis B virus in one patient. These affected patients recovered after anti-viral drugs and Ig administration. Medical history of COVID-19 infection was obtained from 19 surviving patients, of whom six were negative and 13 had been infected during the global pandemic period (years 2020 ∼ 2022). All 13 affected patients presented with self-limiting symptoms, such as low fever, cough, and fatigue. These physical signs lasted from 1 to 3 days variably. No COVID-19-related pneumonia or death was reported. The transgene-specific replication-competent lentivirus had been undetectable in the routine test. Uncontrollable lymphocyte expansion under the circumstance of viral infection was not observed.

To the cut-off date, four patients developed SPM of the lung, esophagus, and cervix at 8 ∼ 32 months after LCAR-B38M treatment. All achieved myeloma remission and had no concurrent hematological malignancies detectable with the SPM. Three patients eventually died of SPM. One patient was cured of cervical cancer but had a MM relapse at 50.0 months post-infusion, and achieved CR after treatment with bortezomib and lenalidomide.

### CAR T cells kinetics

During an observatory period up to 180 days after CAR T cell infusion, the average circulating lymphocyte counts rose to a peak level at 14 ∼ 28 days, and gradually declined after 1 month (Fig. 3F). Though the average lymphocyte count peaks of the non-PD cohort tended to be higher than that of PD/Death patients as CAR T expanded (Fig. 3F), a high peak level of LCAR-B38M did not correlate with CR and durable remission in 16 patients with available transgene data (Supplementary Fig. 4A and 4B). The long duration of the transgene persistence (T_last_) was theoretically relevant to favorable outcome. Patients who achieved very good partial response or better had a longer T_last_ compared with those having PR or worse (421.0 days versus 141.8 days at median, *p* = 0.032). Notably, patients with a longer time to best response had longer CAR T cell persistence compared to those with shorter time to best response (535.3 days versus 261.6 days at median, *p* = 0.001) (Table 3). However, in the long-term observation, long persistence of CAR T cells did not confer a durable remission (Table 3).

**Table 3:** Prognostic relevance of T_last_

Parameters	Evaluable patient number	Mean T_last_ (SD)	*P* value
Best response	53		
VGPR or better	47	421.0 days (389.78)	0.032
PR or worse	6	141.8 days (193.07)	
Time to best response	51		
< 3.3 months	25	261.6 days (389.89)	0.001
≥ 3.3 months	26	535.3 days (333.81)	
Long-term outcome	53		
Durable remission	9	541.1 days (593.63)	0.372
Progression or death	44	358.3 days (324.25)	

*Note* *P* values were calculated by Wilcoxon rank-sum test. PR, Partial response; SD, Standard deviation; T_last_, Transgene persistence; VGPR, Very good partial response

**Fig. 4:**
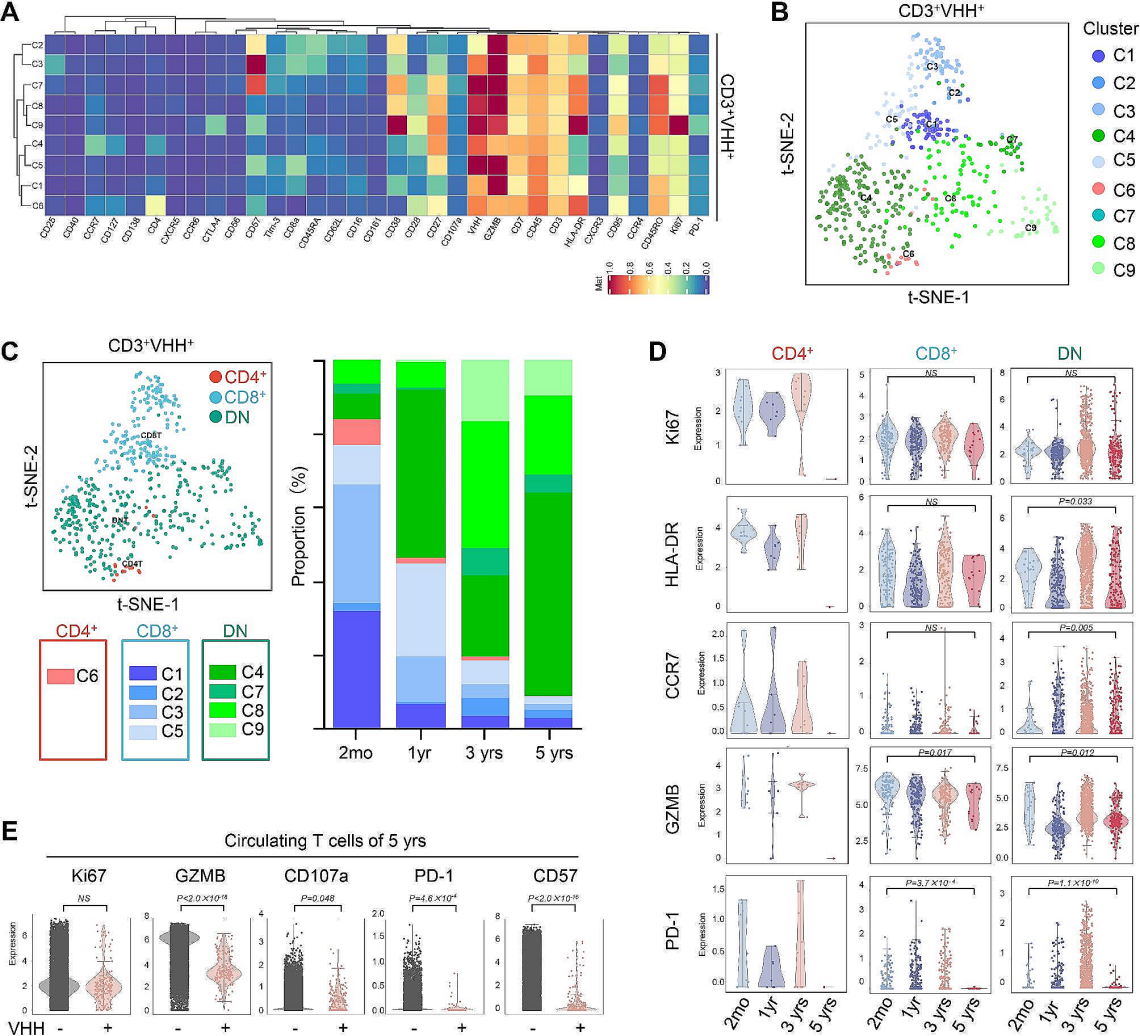
Immunophenotypic characterization of a long-lived CAR T population in a single patient. (**A**) Heatmap shows normalized expression of 33 surface markers of CAR T cells for nine clusters identified with FlowSom. CAR T population was gated upon CD45^+^CD11b^−^CD11c^−^CD66b^−^CD19^−^CD14^−^CD7^+^CD3^+^VHH^+^. Relative frequencies are displayed as a bar graph to the right bottom. (**B**) t-SNE map displays CAR T cells in the four samples of different time points. Cells are colored by FlowSom cluster. (**C**) The left upper panel shows three subsets of CD4^+^, CD8^+^ and DN CAR T cells in the four samples of different time points. The left bottom panel shows the cluster disposition of each CAR T cell subset. The right panel shows the frequencies of 9 clusters in individual sample. DN: CD4 and CD8 double-negative. (**D**) The violin plots display the immunophenotypic expression of Ki67, HLA-DR, CCR7, GZMB and PD-1 of CD4^+^, CD8^+^ and DN CAR T cells of the four samples. *p* values were statistically calculated by comparing the mean marker expression of the sample of 5 years to that of 2 months. (**E**) The violin plots compare the expression levels of Ki67, GZMB, CD107a, PD-1 and CD57 between the CAR T cells (CD3^+^VHH^+^) and the host T cells (CD3^+^VHH^−^). The abbreviations for month and year are mo and yr, respectively

As of the cut-off date, CAR T cells remained detectable in one patient who had maintained MRD-negative CR for 5.6 years at the last follow-up (Supplementary Table 9). The immunophenotype of the long-persisting CAR T cells was examined at 2 months, 1 year, 3 years, and 5 years after infusion using a 41-antibody panel (Supplementary Table 10). CAR T cells labeled by an anti-camelid VHH antibody made up 3.01%, 1.04%, 0.49%, and 0.24% of the CD45^+^ nucleated cells in circulation at the four time points, respectively. Based on the marker expression profile (Fig. 4A and Supplementary Fig. 5), the CAR T cell pool could be assigned into 9 clusters (denoted as C1 ∼ 9) using the algorithm of t-SNE clustering (Fig. 4B and Supplementary Fig. 6A). Signature markers first distinguished three major subsets: CD4^+^ (C6), CD8^+^ (C1 ∼ 3, C5) and CD4^−^CD8^−^ (C4, C7 ∼ 9) (Fig. 4C). Surprisingly, the CD4^−^CD8^−^ subset expanded proportionately with time, accounting for 91.6% of all CAR T cells at 5 years. The CD8^+^ subset was less than 10% and CD4^+^ engineered cells were barely detectable (Fig. 4C). We further found the CD4/CD8 double negative (DN) CAR T subset at later time points exhibited high levels of HLA-DR, CCR7, CD27, CD28 and CD107a, comparable levels of Ki67 and CD38, and low levels of GZMB, PD-1 and Tim-3 as compared to that at initial treatment phase (Fig. 4D and Supplementary Fig. 6B). In the CD8^+^ subset, Ki67 and HLA-DR levels were similar at 2 months and 5 years, but expression of GZMB and PD-1 was decreased at 5 years (Fig. 4D and Supplementary Fig. 6B). Compared with host T cells (CD3^+^VHH^−^), CAR T cells (CD3^+^VHH^+^) featured regular proliferation with Ki67 moderately expressed, functional activation with PD-1 and CD57 down-regulation, and low cytotoxicity with reduced GZMB and CD107a expression at the 5-year time point (Fig. 4E).

## Discussion

We reported the updated efficacy and safety results after a five-year observation of the 74 participants enrolled in the LEGEND-2 trial, which represented the longest follow-up of CAR T cell therapy as a single agent in the treatment of myeloma so far.

Despite advances in MM treatment, the real-world outcomes of the patients with RRMM remain dismal, including the triple-class exposed cohort (refractory to IMiDs, PIs, monoclonal antibodies) who have a median PFS and median OS of 4.6 and 12.4 months [11]. Newer classes of anti-myeloma agents approved in recent years, including selinexor (an inhibitor of exportin 1), Teclistamab (BCMA-redirected bispecific T cell engager), and belantamab mafodotin (an antibody-drug conjugate targeting BCMA), have reported median PFS between 3.7 to 11.3 months [12–14].

Currently, four anti-BCMA CAR T cell products were authorized worldwide for MM, two by the FDA in the USA (idecabtagene vicleucel, cilta-cel) and two by the National Medical Product Administration in China (equecabtagene autoleucel, zevorcabtagene autoleucel). For LCAR-B38M, which uses the same CAR construct as cilta-cel, we previously reported a median PFS of 18 months [7]. The present work demonstrated a median OS of nearly 5 years, with an even longer survival for patients with deep response. Similar outcomes were also observed in the CARTIFAN-1 and CARTITUDE-1 [2, 15]. Importantly, the prognostic results from the indirect comparisons of CARTITUDE-1 with MAMMOTH or KarMMa studies were in favor of cilta-cel compared with real-world therapies and compared with idecabtagene vicleucel [16, 17]. More encouragingly, either indirect or direct comparative studies of cilta-cel in CARTITUDE-4 versus other conventional regimens provided strong evidence of its superiority in earlier lines of treatment [4, 18].

The present work highlights a group of advanced RRMM patients who had survived and maintained durable responses for more than 6 years in the absence of any other anti-myeloma drugs. These patients recovered normal humoral immunity, and we speculate that their myeloma cells have been eradicated. Pleasantly, the majority of them returned to normal life and work. Indeed, a considerable improvement in general health status has been observed in cilta-cel-exposed patients, as well as a physical function and emotional health according to a health-related quality of life investigation of the CARTITUDE-1 trial [19]. CAR T cell treatment, given as a single infusion, changes the conventional concept that effective myeloma treatment is reliant on constant drug interventions.

Depth of response was a key factor associated with durable remission, which aligns with previous work [20]. Of note, an early good response to CAR T, within the initial 3.3 months, did not contribute to a favorable long-term outcome in the current work. Gradual onset of best response conferred a better prognosis. The phenomenon was also reported for anti-CD19 CAR T cells treatment for lymphoma, which was probably related to the substances of the tumor microenvironment [21, 22]. In the myeloma setting, monoclonal immunoglobulins degrade over several days or weeks, contributing to a late CR. The half-life of IgG is the longest, followed by IgA and IgD. This could explain the reason patients who had a longer time to reach the best response were more likely belonging to IgG subtype disease. Other positive factors including good performance status, absence of extramedullary involvement and addition of fludarabine in the preconditioning chemotherapy were consistently deemed as determinants beneficial to patients’ outcomes [23, 24].

CAR T cells with sustained persistence and mild proliferative capacity could potentially play a role in long-term tumor surveillance. In the context of hematological malignancy, the longest persistence of CAR T cells, thus far, was reported in two chronic lymphoid leukemia (CLL) patients who had detectable circulating anti-CD19 engineered cells for over 10 years post-infusion. In those two cases, the long-persisting adoptively transferred cells were enriched with CD4^+^ subpopulation possessing functional activity such as cytotoxic effect [25]. Another study revealed the long-lived anti-CD19 CAR T cells in pediatric acute lymphoblastic leukemia (ALL) patients developed a CD4/CD8 DN phenotype with an exhausted-like memory state [26]. The long-lived cells in our LEGEND-2 patient resembled the phenotype observed in the childhood ALL cases, with a predominantly CD4/CD8 DN phenotype in the later years. DN lymphocytes, a low-frequency subpopulation of the immune system, take part in inflammation regulation, pathogenic clearance and anti-tumor response [27, 28]. As the host lymphocytes did not have a detectable DN subset, DN engineered cells are speculated to arise from a CAR T cell with CD4 or CD8 expression [28]. Importantly, the long-lived CAR T cells did not show aggressive expansion capability but did have active T lymphocyte functionality, presumably serving a tumor immune surveillance function. Alternatively, given that DN T lymphoma cells were reported in several cases with mycosis fungoides [29, 30], a possible malignant transformation of the persisting CAR T cells remains a risk that cannot be completely ignored and requires close watching.

It is worth noting that disease recurrence still happens after 5-year clinical remission. Tumor re-emergence after a period of MRD-negative CR is attributable to residual MM cells below the limits of flow cytometry sensitivity and imaging resolution. Thus, maintenance therapy following CAR T treatment for a durable response should be introduced before myeloma relapse. There are three key questions to address: (1) when to give maintenance therapy? (2) who should receive it? (3) which approaches are the most appropriate? Usually, subsequent anti-myeloma therapy including either a previously used drug or a new regimen is initiated after progression on CAR T treatment. In reality, a portion of patients still could obtain response benefit [31], which was consistent with what we observed in the LEGEND-2 participants. Among the accessible approaches, CAR T cell re-treatment is still worth trying. The benefit of re-treatment of anti-BCMA CAR T cells has been demonstrated by the real-world experience of idecabtagene vicleucel [32]. CAR T cell against GPRC5D is also an alternative [33]. The potential effectiveness of other BCMA-directed immunotherapies including Teclistamab and belantamab mafodotin needs more supporting clinical evidence. Prospectively, CAR T-based combinatory scheme can be explored for prompting a synergistic effect, such as CAR T combined with selinexor in the treatment of the patients with extramedullary lesion or plasmacytoma [34].

In the present work, all SPM were solid tumors with an incidence of 5.4%. This incidence was lower than the recent statistical results from a large cohort analysis of adult patients, in which, the 5-year estimated incidence of second solid tumor was 15.2% and the median onset was 2.2 years after commercial CAR T [35]. In young patients, the rate of solid neoplasm after CD19-redirected CAR T therapy was relatively low (∼ 1%) with the median time to SPM appearance being 3.2 years [36]. Age above 65 is an independent risk factor for SPM development after CAR T therapy, irrespective of gender, lines of prior therapy, and CAR construct [35], implicating age-related genomic instability in tumorigenesis. Therefore, it is reasonable that patients with diseases that arise later in life, such as CLL and MM, are more susceptible to somatic mutagenesis. This has been observed by both our previous study and that of Fraietta’s study [37, 38], in which, acquired *TET2* mutations identified in elderly MM and CLL promoted CAR T expansion. Indeed, the four MM patients in this work respectively developed SPM at the age of 73, 68, 63, and 52 years old, consistent with age as a key risk factor for SPM. Given these observations, administration of CAR T in earlier lines of treatment, reduction of genotoxic exposure, and monitoring for SPM are warranted in MM.

## Conclusions

LCAR-B38M as a monotherapy achieved long-term remission in a group of intensively pretreated patients with RRMM, and 16% of the study population remain relapse-free after > 5 years. This advancement in improved outcomes for patients with MM has the potential to reshape the anti-myeloma therapeutic landscape and raises the possibility of a cure in a subset of patients.

### Electronic supplementary material

Below is the link to the electronic supplementary material.

Supplementary Material 1

## Data Availability

Access to study data may be requested from the corresponding authors.

## Abbreviations

*ALL*: acute lymphoblastic leukemia
*BCMA*: B-cell maturation antigen
*BM*: bone marrow
*CA*: cytogenetic abnormalities
*CAR*: chimeric antigen receptor
*CI*: confidence interval
*Cilta-cel*: ciltacabtagene autoleucel
*CLL*: chronic lymphoid leukemia
*CR*: complete response
*CRS*: cytokine release syndrome
*CZ*: Changzheng Hospital
*DN*: CD4/CD8 double negative
*DOR*: duration of response
*ECOG*: Eastern Cooperative Oncology Group
*Ig*: immunoglobulin
*IMiD*: immunomodulatory drug
*ISS*: International Staging System
*MM*: multiple myeloma
*MRD*: measurable residual disease
*NJ*: First Affiliated Hospital of Nanjing Medical University
*ORR*: overall response rate
*OS*: overall survival
*PD*: progressive disease
*PFS*: progression-free survival
*PI*: proteasome inhibitor
*PR*: partial response
*RJ*: Ruijin Hospital affiliated with Shanghai Jiao Tong University
*RRMM*: relapsed and refractory multiple myeloma
*SPM*: second primary malignancy
T_last_: transgene persistence
*TEAE*: treatment-emergent adverse event
*USA*: United States of America
*VHH*: variable domain of heavy chain of heavy-chain
*Xi’an*: Second Affiliated Hospital of Xi’an Jiao Tong University
