# Supplementary material for: Long-term remission and survival in patients with relapsed or refractory multiple myeloma after treatment with LCAR-B38M CAR T cells: 5-year follow-up of the LEGEND-2 trial
Source: J Hematol Oncol. 2024 Apr 24;17:23. doi: 10.1186/s13045-024-01530-z (PMC11040812; doi:10.1186/s13045-024-01530-z)
Supplement: Supplementary file 1 — Supplementary Material 1 [file 13045_2024_1530_MOESM1_ESM.pdf]

Supplementary Data

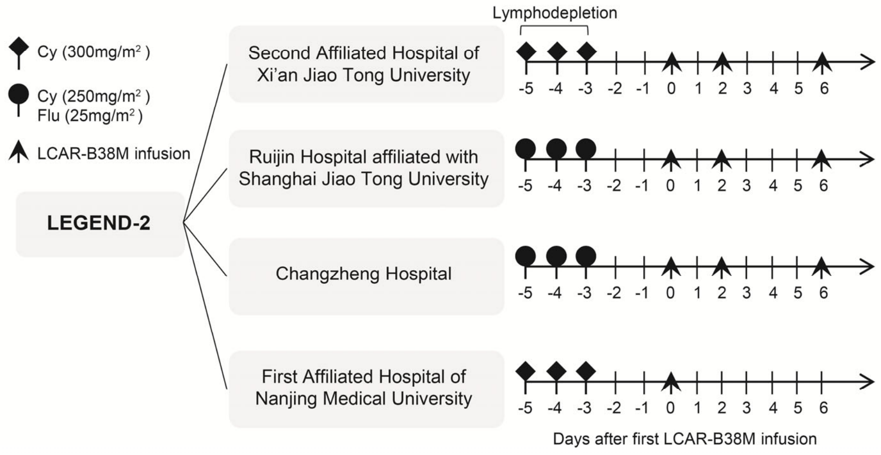

Supplementary Figure 1. Treatment scheme of LCAR-B38M

The schematic illustration displays the lymphodepletion regimens and LCAR-B38M infusion modes adopted by four different participating centers of the LEGEND-2 trial. Cy: Cyclophosphamide; Flu: Fludarabine.

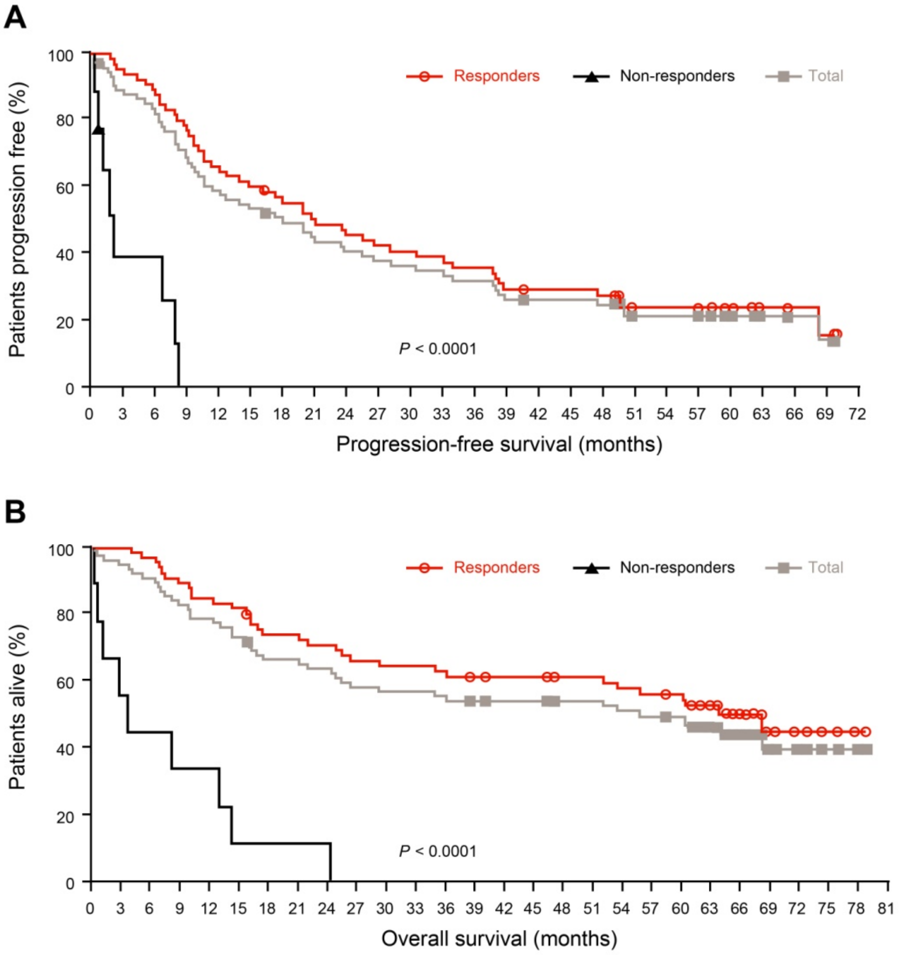

Supplementary Figure 2. Long-term efficacy of the responders to LCAR-B38M treatment

(**A, B**) The Kaplan-Meier survival curves compare the progression-free survival (**A**) and overall survival (**B**) rates between the 65 patients who responded to CAR T cell therapy (red) and the rest 9 patients (black) who were resistant to LCAR-B38M or had early deaths.

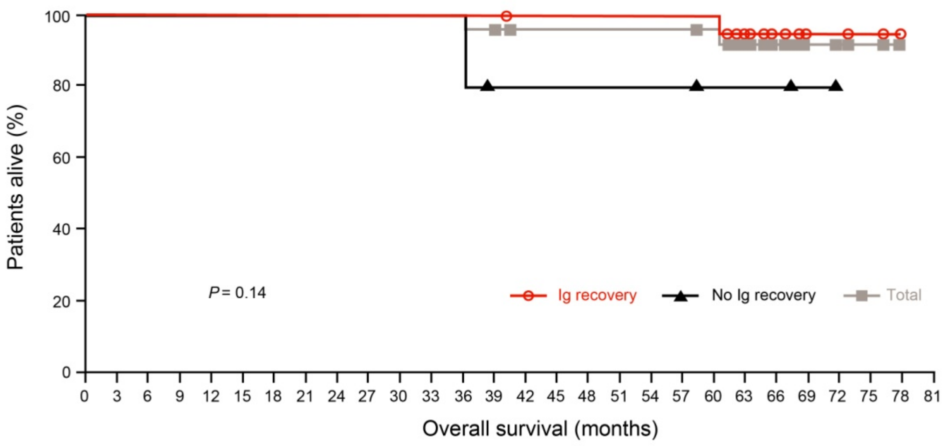

Supplementary Figure 3. Outcomes of the complete responders with or without serum immunoglobulin recovery

This analysis included 26 patients who achieved complete responses (CR) and had available serological immunoglobulin (Ig) reports in the follow-up. The Kaplan-Meier survival curves show the overall survival rates of the CR patients gaining serum Ig recovery (red) or not (black).

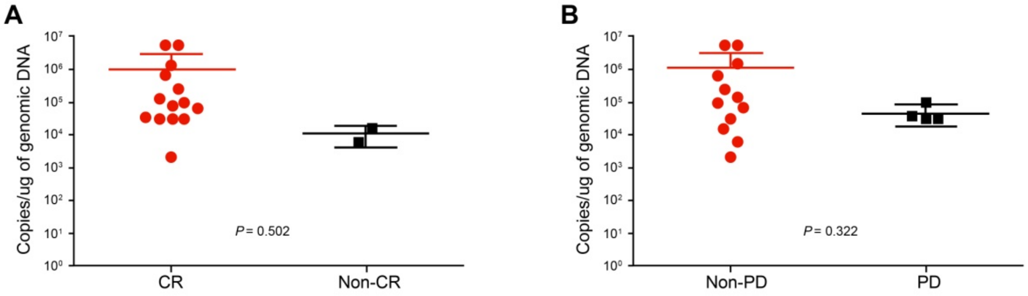

Supplementary Figure 4. Correlation of patient outcome and LCAR-B38M expansion level

There were 16 patients had transgenes detected by quantitative PCR technique across LCAR-B38M treatment. (**A**) The graph shows the peak levels of the transgene copy numbers of the 14 patients who ultimately achieved complete responses (CR) and two who did not (Non-CR). The results were shown as mean±SEM. *p* value was calculated by *t*-test. (**B**) The column plots show the peak levels of the transgene copy numbers of the 4 patients without experiencing disease progression (Non-PD) and the remaining 12 who had progressed disease (PD). The data were displayed as mean± SEM. *p* value was calculated by *t*-test.

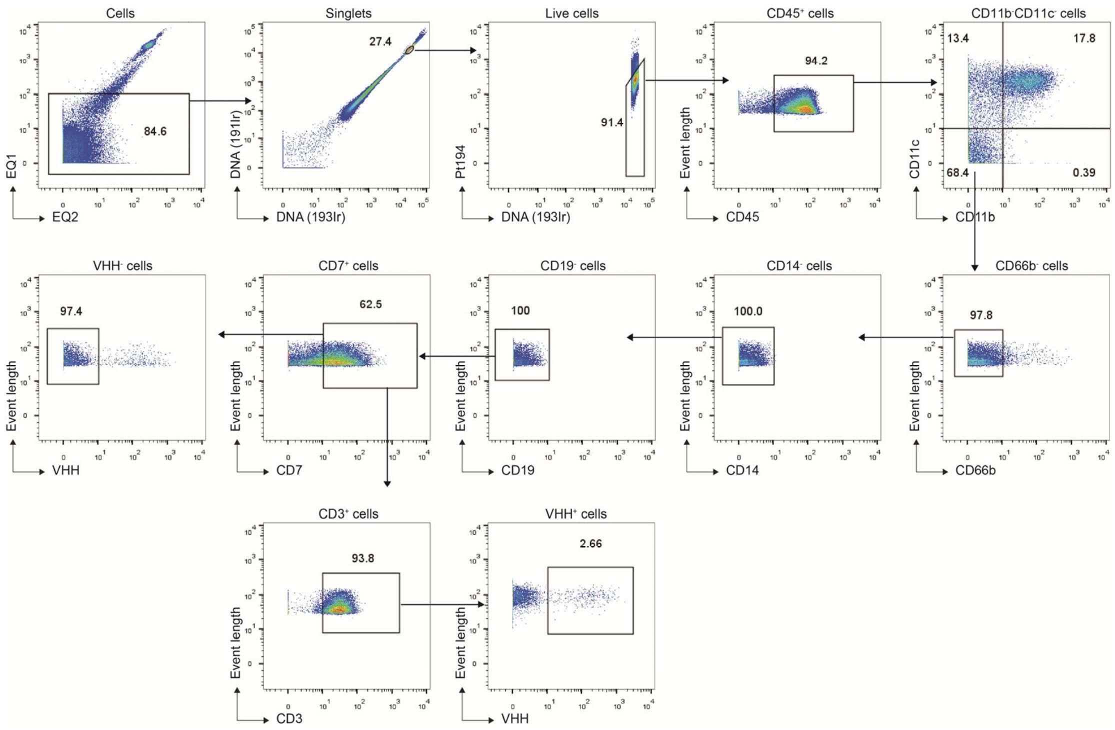

Supplementary Figure 5. Gating strategy of mass cytometry data analysis

The representative plots show the gating strategy to access the T lymphocyte population, by which, the single, living nucleated cells were initially selected, followed by the removal of granulocytes ($CD11b^+$ and/or $CD11c^+$, and $CD66b^+$), monocytes ($CD14^+$) and B lymphocytes ($CD19^+$). Subsequently, based on $CD7^+$ cells gating, $CD3^+VHH^+$ population regarded as CAR T cells was chosen for further investigation. VHH: a purified camelid-heavy-chain-antibody antibody to label LCAR-B38M.

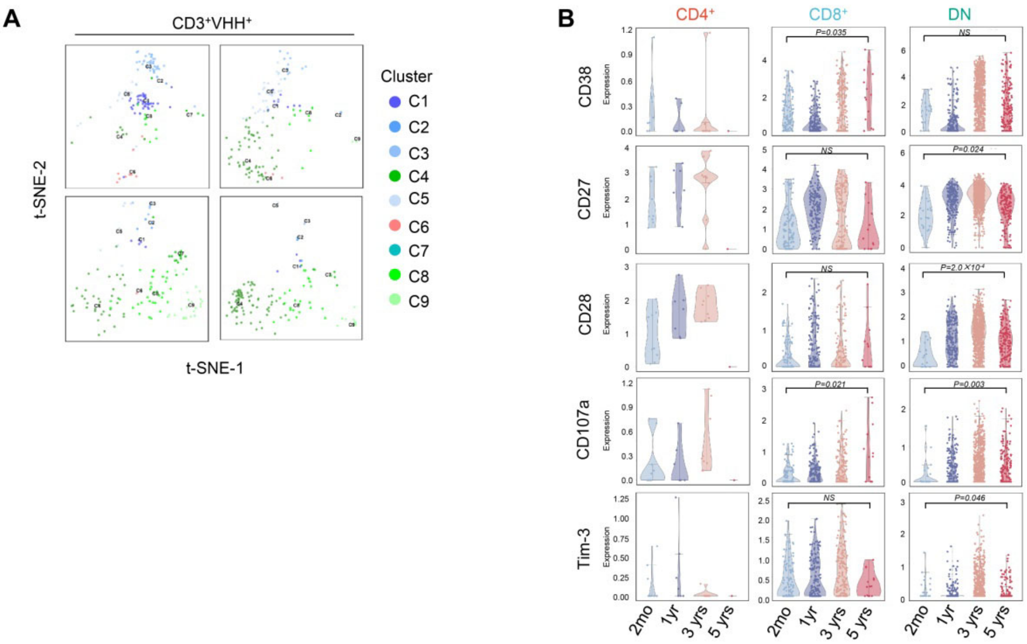

Supplementary Figure 6. Immunophenotypic characterization of the CAR T population persistently present in one case

(**A**) t-SNE maps show the different cluster compositions of the CAR T populations at different time points. Clusters were defined as C1~9. (**B**) The violin plots display the immunophenotypic expression of CD38, CD27, CD28, CD107a and Tim-3 of $CD4^+$, $CD8^+$ and double negative (DN) CAR T cells of the four samples. *p* values were statistically calculated by comparing the mean marker expression of the sample of 5 years to that of 2 months. The abbreviations for month and year are mo and yr, respectively.

Supplementary Table 1. Prior therapies of the patients in the LEGEND-2 study

PIN	Sex	Age	MM type	PI	IMiD	Alkylating agent (Y/N)	Anthracycline (Y/N)	Auto-HSCT (Y/N)
1-001	M	61	IgA,κ	No	Thalidomide	Y	Y	N
1-002	F	50	IgA,λ	No	Thalidomide	Y	Y	N
1-003	F	51	IgG,κ	No	Thalidomide	Y	Y	N
1-004	M	57	IgG,λ	Bortezomib	Thalidomide	N	N	N
1-005	F	49	λ	Bortezomib	Thalidomide	Y	Y	N
1-006	M	56	λ	No	Lenalidomide	Y	Y	N
1-007	F	57	λ	Bortezomib	Thalidomide	Y	Y	N
1-008	M	48	IgG,λ	Bortezomib	Thalidomide Lenalidomide	Y	Y	N
1-009	F	53	IgG,λ	Bortezomib	No	Y	Y	N
1-010	M	46	IgG,κ	Bortezomib	Lenalidomide	Y	Y	Y
1-011	M	44	IgA,λ	No	Thalidomide	Y	Y	N
1-012	F	71	IgA,κ	Bortezomib	No	Y	Y	N
1-013	M	66	λ	No	Thalidomide	Y	Y	N
1-014	F	46	IgG,λ	Bortezomib	No	Y	Y	Y
1-015	M	51	IgG,λ	Bortezomib	Lenalidomide	N	N	Y
1-016	M	57	IgA,κ	No	No	Y	Y	N
1-017	F	49	IgG,κ	Bortezomib	No	Y	Y	N
1-018	F	46	IgA,κ	Bortezomib	Thalidomide Lenalidomide	Y	Y	Y
1-019	M	66	IgA,κ	No	Thalidomide Lenalidomide	Y	Y	N
1-020	M	68	λ	Bortezomib	Thalidomide	N	N	N
1-021	F	61	IgG,λ	Bortezomib	Thalidomide	Y	Y	N
1-022	M	53	λ	Bortezomib	Thalidomide	Y	Y	N
1-023	F	50	IgG,κ	No	Thalidomide	Y	Y	N
1-024	M	68	IgG,κ	Bortezomib	Lenalidomide	Y	Y	N
1-025	M	52	IgG,κ	No	No	Y	Y	N
1-026	F	67	λ	No	Thalidomide	Y	Y	N
1-027	F	48	IgA,κ	Bortezomib	Thalidomide Lenalidomide	N	Y	Y
1-028	M	60	IgA,κ	Bortezomib	Thalidomide Lenalidomide	Y	Y	N
1-030	M	49	IgG,λ	No	No	Y	Y	N
1-031	F	63	IgG,κ	No	Thalidomide	Y	Y	N
1-032	M	53	κ	No	Thalidomide	Y	Y	N
1-034	F	43	IgA,κ	Bortezomib	Thalidomide	Y	Y	Y

1-035	M	65	κ	Bortezomib	No	Y	N	N
1-036	F	60	IgG,λ	Bortezomib	Thalidomide	Y	N	N
1-037	M	47	κ	No	Thalidomide Lenalidomide	Y	Y	N
1-038	M	51	IgG,λ	Bortezomib	Thalidomide	Y	Y	N
1-039	M	55	IgA,λ	Bortezomib	Thalidomide	N	N	N
1-042	F	65	κ	Bortezomib	Thalidomide	Y	Y	N
1-046	M	45	IgG,λ	Bortezomib	Thalidomide Lenalidomide	Y	Y	N
1-047	F	72	IgA,κ	Bortezomib	Thalidomide Lenalidomide	Y	Y	N
1-049	M	48	IgA,κ	No	Thalidomide	Y	Y	N
1-052	M	48	IgG,λ	Bortezomib	Thalidomide Lenalidomide	Y	N	N
1-053	M	63	IgG,κ	Bortezomib	Thalidomide Lenalidomide	Y	N	N
1-055	M	62	λ	Bortezomib	Lenalidomide	Y	N	N
1-056	M	65	IgG,λ	No	Thalidomide	Y	Y	N
1-057	M	67	IgA,κ	Bortezomib	Thalidomide Lenalidomide	Y	Y	N
1-058	M	52	IgG,λ	Bortezomib	Lenalidomide	Y	Y	N
1-059	F	54	κ	No	Thalidomide	Y	Y	N
1-060	M	55	IgG,κ	Bortezomib	Thalidomide Lenalidomide	Y	Y	N
1-061	M	64	IgG,λ	Bortezomib	Thalidomide Lenalidomide	Y	Y	N
1-063	F	54	IgA,κ	Bortezomib	Lenalidomide Pomalidomide	Y	N	Y
1-066	F	55	IgG,κ	Bortezomib	Lenalidomide Pomalidomide	Y	N	N
1-068	M	27	κ	Bortezomib	Thalidomide	Y	Y	Y
1-071	F	44	κ	Bortezomib	Lenalidomide	Y	N	Y
1-072	F	55	λ	Bortezomib	Thalidomide Lenalidomide	N	Y	N
1-073	M	54	λ	Bortezomib	Lenalidomide	Y	Y	Y
1-074	M	48	IgG,λ	Bortezomib Carfilzomib	Thalidomide Lenalidomide	Y	Y	N
2-033	F	61	IgG,λ	Bortezomib	Lenalidomide	Y	Y	Y

2-041	M	57	IgD,λ	Bortezomib	Lenalidomide	Y	Y	N
2-043	M	55	IgG,κ	Bortezomib	Lenalidomide	Y	Y	N
2-069	M	68	IgG,κ	Carfilzomib	No	Y	N	N
2-075	F	56	IgG,λ	No	Lenalidomide	Y	Y	N
3-040	M	35	IgG,κ	No	Thalidomide	Y	Y	Y
3-044	F	47	IgG,λ	Bortezomib	Thalidomide<br>Lenalidomide	Y	Y	Y
3-065	F	40	IgG,λ	Bortezomib	No	Y	N	N
4-029	M	67	IgA,κ	Bortezomib	Thalidomid	Y	Y	N
4-045	M	74	λ	Bortezomib	Thalidomide<br>Lenalidomide	Y	N	N
4-048	M	63	IgA,κ	Bortezomib	Thalidomide<br>Lenalidomide	Y	Y	N
4-050	M	52	λ	Bortezomib	No	Y	N	Y
4-051	F	53	IgG,κ	Bortezomib	Thalidomide	Y	Y	Y
4-054	M	56	IgA,κ	Bortezomib<br>Carfilzomib<br>Ixazomib	Lenalidomide<br>Pomalidomide	Y	Y	Y
4-062	F	63	IgA,κ	Bortezomib	Lenalidomide	Y	Y	Y
4-067	M	53	IgA,κ	Bortezomib	Lenalidomide	N	N	Y
4-076	M	35	IgA,κ	Bortezomib	Thalidomide	Y	Y	N

Abbreviation: HSCT, hematopoietic stem cell transplantation; IMiD, immunomodulatory drug; MM, multiple myeloma; PI, proteasome inhibitor; PIN, patient identification number.

Supplementary Table 2. Progression-free survival of the patients with or without CR

	Patients with CR	Patients without CR	Total
All-treated Analysis Set	54	20	74
Progresional-free Survival			
Number of events (%)	38 (70.4%)	19 (95.0%)	57 (77.0%)
Number of censored (%)	16 (29.6%)	1 (5.0%)	17 (23.0%)
Kaplan-Meier estimate (months)			
25% quantile (95% CI)	13.96 (9.63, 19.98)	1.97 (0.43, 3.22)	7.95 (5.16, 9.72)
Median (95% CI)	28.16 (19.98, 38.74)	4.44 (1.97, 7.85)	18.04 (10.61, 26.58)
75% quantile (95% CI)	68.27 (38.28, NE)	8.25 (4.44, 9.20)	47.44 (30.55, NE)
p value [a]		<0.0001	

Abbreviation: CI, confidence interval; CR, complete response.

Note: if the patient had only month and year available for the death date, disease progression date, subsequent therapy date, day 15 is used for imputation.

[a] *p* value is based on the log-rank test.

Supplementary Table 3. Progression-free survival of the patients with or without MRD-negative CR

	Patients with MRD-negative CR	Patients without MRD-negative CR	Total
All-treated Analysis Set	50	24	74
Progresional-free survival			
Number of events (%)	35 (70.0%)	22 (91.7%)	57 (77.0%)
Number of censored (%)	15 (30.0%)	2 (8.3%)	17 (23.0%)
Kaplan-Meier estimate (months)			
25% quantile (95% CI)	14.95 (10.61, 21.06)	2.14 (0.43, 4.44)	7.95 (5.16, 9.72)
Median (95% CI)	30.55 (20.76, 47.44)	6.44 (2.33, 8.25)	18.04 (10.61, 26.58)
75% quantile (95% CI)	68.27 (38.74, NE)	9.10 (6.77, 10.12)	47.44 (30.55, NE)
p value [a]		<0.0001	

Abbreviation: CI, confidence interval; CR, complete response; MRD, minimal residual disease.

Note: if the patient had only month and year available for the death date, disease progression date, subsequent therapy date, day 15 is used for imputation.

[a] *p* value is based on the log-rank test.

Supplementary Table 4. Overall survival of the patients with or without CR

	Patients with CR	Patients without CR	Total
All-treated Analysis Set	54	20	74
Overall survival			
Number of events (%)	22 (40.7%)	19 (95.0%)	41 (55.4%)
Number of censored (%)	32 (59.3%)	1 (5.0%)	33 (44.6%)
Kaplan-Meier estimate (months)			
25% quantile (95% CI)	29.27 (16.20, 60.42)	4.01 (0.43, 6.90)	14.23 (7.49, 21.13)
Median (95% CI)	NE (60.29, NE)	7.87 (3.84, 14.23)	55.79 (24.44, NE)
75% quantile (95% CI)	NE (NE, NE)	19.33 (8.25, 53.45)	NE (NE, NE)
p value [a]		<0.0001	

Abbreviation: CI, confidence interval; CR, complete response.

Note: if the patient had only month and year available for the death date, day 15 is used for imputation.

[a] *p* value is based on the log-rank test.

Supplementary Table 5. Overall survival of the patients with or without MRD-negative CR

	Subjects with MRD-negative CR	Subjects without MRD-negative CR	Total
All-treated Analysis Set	50	24	74
Overall survival			
Number of events (%)	20 (40.0%)	21 (87.5%)	41 (55.4%)
Number of censored (%)	30 (60.0%)	3 (12.5%)	33 (44.6%)
Kaplan-Meier estimate (months)			
25% quantile (95% CI)	34.96 (16.89, 60.42)	4.67 (0.43, 7.06)	14.23 (7.49, 21.13)
Median (95% CI)	NE (60.29, NE)	9.23 (5.16, 16.30)	55.79 (24.44, NE)
75% quantile (95% CI)	NE (NE, NE)	24.69 (12.42, NE)	NE (NE, NE)
p value [a]		<0.0001	

Abbreviation: CI, confidence interval; CR, complete response; MRD, minimal residual disease.

Note: if the patient had only month and year available for the death date, day 15 is used for imputation.

[a] *p* value is based on the log-rank test.

Supplementary Table 6. Duration of response of the patients with or without MRD-negative CR

	Subjects with MRD-negative CR	Subjects without MRD-negative CR	Total
All-treated Analysis Set	50	15	65
Duration of response			
Number of events (%)	33 (66.0%)	13 (86.7%)	46 (70.8%)
Number of censored (%)	17 (34.0%)	2 (13.3%)	19 (29.2%)
Kaplan-Meier estimate (months)			
25% quantile (95% CI)	14.32 (9.46, 23.26)	2.17 (0.36, 5.72)	8.80 (5.72, 11.79)
Median (95% CI)	32.69 (22.34, 49.05)	7.46 (2.00, 8.61)	23.26 (13.04, 36.50)
75% quantile (95% CI)	67.65 (45.54, NE)	8.61 (5.72, NE)	67.65 (36.50, NE)
p value [a]		<0.0001	

Abbreviation: CI, confidence interval; CR, complete response; MRD, minimal residual disease.

[a] *p* value is based on the log-rank test.

Supplementary Table 7. Correlation of time to best response with patient basic characteristics

	Time to best response <3.3 months (n=32)	Time to best response ≥3.3 months (n=33)	Total (n=65)	*p* value
Sex, n (%)				
Male	21 (65.6)	19 (57.6)	40 (61.5)	0.5049
Female	11 (34.4)	14 (42.4)	25 (38.5)	
Age, years (range)	55.5 (27~74)	52 (35~71)	54 (27~74)	0.3353
Performance status at baseline, n (%)				
0	14 (43.8)	14 (42.4)	28 (43.1)	
1	11 (34.4)	16 (48.5)	27 (41.5)	0.2849
2	7 (21.9)	3 (9.1)	10 (15.4)	
Type of myeloma by immunofixation, n (%)				
IgG	8 (25.0)	21 (63.6)	29 (44.6)	0.0017
IgA	14 (43.8)	6 (18.2)	20 (30.8)	0.0256
IgD	1 (3.1)	0	1 (1.5)	0.4923
Kappa	5 (15.6)	1 (3.0)	6 (9.2)	0.1048
Lambda	4 (12.5)	5 (15.2)	9 (13.8)	1.0000
Disease Stage: Durie-Salmon system, n (%)				
IIA	4 (12.5)	4 (12.1)	8 (12.3)	
IIB	1 (3.1)	0	1 (1.5)	
III	2 (6.3)	0	2 (3.1)	0.7424
IIIA	19 (59.4)	20 (60.6)	39 (60.0)	
IIIB	3 (9.4)	4 (12.1)	7 (10.8)	
Unknown	3 (9.4)	5 (15.2)	8 (12.3)	
ISS Stage, n (%)				
I	17 (53.1)	13 (39.4)	30 (46.2)	
II	2 (6.3)	10 (30.3)	12 (18.5)	0.0491
III	10 (31.3)	8 (24.2)	18 (27.7)	
Unknown	3 (9.4)	2 (6.1)	5 (7.7)	

Abbreviation: ISS, International Staging System

Note: *t*-test was used for comparing the difference in continuous data. Chi-square test was performed for discrete data. When Chi-square test was not appropriate, Fisher's exact test was used instead.

Supplementary Table 8. Normal immunoglobuline recovery in the 21 patients with complete responses

Parameters	Non-PD	PD/Death	Total
Number of patients	12	9	21
Number of yearly recovery (n)			
1-year recovery	1 (8.3%)	2 (4.8%)	3 (5.6%)
2-year recovery	7 (58.3%)	8 (19.0%)	15 (27.8%)
3-year recovery	8 (66.7%)	8 (19.0%)	16 (29.6%)
4-year recovery	11 (91.7%)	9 (21.4%)	20 (37.0%)
5-year recovery	11 (91.7%)	9 (21.4%)	20 (37.0%)
6-year recovery	12 (100.0%)	9 (21.4%)	21 (38.9%)
Time to recovery (months)			
Mean (SD)	27.6 (16.4)	17.8 (10.7)	23.4 (14.8)
Median (range)	22.4 (9.1~65.3)	15.2 (12.0~45.6)	16.7 (9.1~65.3)
κ/λ ratio at recovery			
Mean (SD)	1.6 (0.8)	1.8 (0.4)	1.7 (0.7)
Median (range)	1.8 (0.4~2.7)	1.8 (0.8~2.5)	1.8 (0.4~2.7)

Abbreviation: PD, progressive disease; SD, standard deviation.

Supplementary Table 9. Patients with PFS longer than 5 years

PIN	First Infusion date	PFS duration (year)	Disease evaluation	Survival status	CAR T detectable in PB
1-006	2016/6/4	5.7	Disease Progression*	Alive	No
1-009	2016/7/25	6.4	CR	Alive	No
1-011	2016/11/3	6.1	CR	Alive	No
1-021	2017/2/9	5.8	Disease Progression*	Alive	No
1-024	2017/2/21	5.8	CR	Alive	No
1-025	2017/3/6	5.7	CR	Alive	No
1-027	2017/3/25	5.7	CR	Alive	No
1-038	2017/6/12	5.5	CR	Alive	No
1-039	2017/6/16	5.5	CR	Alive	No
1-058	2017/8/14	5.3	CR	Alive	No
2-033	2017/5/3	5.6	CR	Alive	Yes
2-069	2017/9/19	5.2	CR	Alive	No
2-075	2017/10/24	5.1	CR	Alive	No
3-040	2017/6/16	5.4	CR	Alive	No
4-062	2017/8/29	5.2	Disease Progression*	Alive	No

Abbreviation: CAR, chimeric antigen receptor; PB, peripheral blood; PFS, progression-free survival; PIN, patient identification number.

Note: CR indicates complete response with flow-based measurable residual disease negative.

*Using month as the time unit, the PFS for the three relapsed patients were 68.3 (1-006), 69.5 (1-021), and 62.4 (4-062) months, respectively.

Supplementary Table 10. The list of 41 antibodies used for mass cytometry assay

Conjugate	Antibody	Source	Identifier
89Y	CD45 (HI30) - purified	BioLegend	Cat# 304002
115ln	CD3 (UCHT1) - purified	Bio Cell	Cat# BE0231
141Pr	CD56 (NCAM16.2) - purified	BD Biosciences	Cat# 559043
142Nd	CD194/CCR4 (L291H4) - purified	BioLegend	Cat# 359402
143Nd	CD196/CCR6 (G034E3) - purified	BioLegend	Cat# 353402
144Nd	CD28 (CD28.2) - purified	BioLegend	Cat# 302934
145Nd	Ki67 (Ki-67) - purified	BioLegend	Cat# 350502
146Nd	CD38 (HIT2) - purified	BioLegend	Cat# 303502
147Sm	CD183/CXCR3 (G025H7) - purified	BioLegend	Cat# 353750
148Nd	CD19 (HIB19) - purified	BioLegend	Cat# 302268
149Sm	CD95/Fas (DX2) - purified	BioLegend	Cat# 305656
150Nd	CD14 (M5E2) - purified	BioLegend	Cat# 301862
151Eu	CD107a/LAMP-1 (H4A3) - purified	BioLegend	Cat# 328602
152Sm	CD27 (O323) - purified	BioLegend	Cat# 302802
153Eu	Camelid VHH Antibody - purified	Genscript	Cat# A01860
154Sm	CD197/CCR7 (G043H7) - purified	BioLegend	Cat# 353222
155Gd	CD185/CXCR5 (RF8B2) - purified	BD Biosciences	Cat# 552032
156Gd	CD11c (BU15) - purified	BioLegend	Cat# 337202
157Gd	CD123/IL-3Rα (6H6) - purified	BioLegend	Cat# 306002
158Gd	CD127/IL-7Rα (A019D5) - purified	BioLegend	Cat# 351302
159Tb	CD45RO (UCHL1) - purified	BioLegend	Cat# 304202
160Gd	CD161 (HP-3G10) - purified	BioLegend	Cat# 339902
161Dy	CD152/CTLA-4 (14D3) - purified	eBioscience	Cat# 14-1529-82
162Dy	CD1c (L161) - purified	BioLegend	Cat# 331502
163Dy	CD138 (DL-101) - purified	BioLegend	Cat# 352311
164Dy	CD141 (M80) - purified	BioLegend	Cat# 344102
165Ho	CD66b (G10F5) - purified	BioLegend	Cat# 305102
166Er	CD45RA (HI100) - purified	BioLegend	Cat# 304102
167Er	CD25 (24212) - purified	R&D Systems	Cat# MAB1020
168Er	CD57 (HNK-1) - purified	BioLegend	Cat# 359602
169Tm	CD40 (82111) - purified	R&D Systems	Cat# MAB6321
170Er	CD7 (CD7-6B7) - purified	BioLegend	Cat# 343102
171Yb	CD279/PD-1 (EH12.2H7) - purified	BioLegend	Cat# 329926
172Yb	CD62L (DREG-56) - purified	BioLegend	Cat# 304812
173Yb	CD366/Tim-3 (F38-2E2) - purified	BioLegend	Cat# 345004
174Yb	Granzyme B (QA16A02) - purified	BioLegend	Cat# 372202
175Lu	CD16 (3G8) - purified	BioLegend	Cat# 302014
176Yb	HLA-DR (L243) - purified	BioLegend	Cat# 307648
197Au	CD4 (RPA-T4) - purified	BioLegend	Cat# 300570
198Pt	CD8a (RPA-T8) - purified	BioLegend	Cat# 301074
209Bi	CD11b (M1/70) - purified	BioLegend	Cat# 101202
